# Neurology and the new decade

**DOI:** 10.4103/0972-2327.61268

**Published:** 2010

**Authors:** Sanjeev V. Thomas

**Affiliations:** Department of Neurology, Sree Chitra Tirunal Institute for Medical Sciences and Technology, Trivandrum, India. E-mail: editor@annalsofian.org

Greetings to all readers as we move in to the next decade in this millennium. Let us hope to witness several exciting developments in the field of neurosciences that would pave way to the cure of many neurological disorders in this new decade. This year the Annals of Indian Academy of Neurology is entering the 13th year of publication. We hope to bring to you some of these excitements. There had been much progress in the field of epileptology. Dr H V Srinivas, in his Presidential Oration has summarized these developments and has challenged us to meet the future scenario, particularly in the realm of treatment and prevention. There is much research going on to improve the techniques of seizure prediction and stimulation techniques to abort seizures. Some of these methodologies are very promising and are undergoing various stages of clinical trials.

Stroke is currently one of the thrust areas of research all across the world. In this issue we carry a review article on optimal secondary prevention of stroke. Patients at higher risk of stroke tend to meet neurologists mostly after the first stroke, although it would be desirable that they are evaluated even before the first stroke. Traditionally, secondary prophylaxis of stroke consists of reducing risk factors for stroke and institution of anti-platelet therapy or antithrombotic therapy. There is much debate about the choice of first antiplatelet, precise dosage of these drugs, and the role of newer antiplatelet drugs. The author has discussed in detail the merits of individual medications and the cumulative evidence base to support its use as an antiplatelet drug. Another issue in this regard is the appropriate use of these medications in special situations such as pregnancy, peripartum period, perioperative period, and in the setting of coronary artery disease or arterial dissection. Dr Mishra and Dr Khadilkar have expressed their view points on what practical steps can be take to improve the of stroke services in this country, especially in view of the vast shortage of neurologists and other experts trained in the management of stroke. In another article on stroke, the authors have addressed the issue of cognitive decline following stroke. In a prospective study, they had observed that more than 30% of stroke survivors had impairment of cognitive functions.

Magnetoencephalography has left the research laboratories and has found more clinical application in the past decade. One of the technical challenges in this field was in designing appropriate technology to isolate the tiny fluxes in the magnetic fields generated from the brain from the changes in the background. The magnetic field of cerabal activitiy is of the order of a few femto tesla (10^−15^), whereas that of the earth is 10^9^ times higher (30 to 60 microtesla). The inventions of superconducting quantum interference devices (SQUID) and magnetically shielded rooms have resolved this problem. One of the limitations of SQUID is the obligatory need to have near absolute zero degree Kevin temperature (−291) which is achieved with the liquid helium. Researchers are now attempting to make optical magnetometers that work on the basis magnetic resonance of atomic vapor. The advantage of optical magnetometers is that it can work at room temperature and is likely to be less expensive and less demanding on maintenance. We have included a comprehensive review article in this issue deals with the clinical application of magnetoencephalography in epilepsy. We hope the readers will find these articles and others informative and useful.

This issue onward, we are advancing the actual date of publication of the issues to the beginning of the quarter. This would bring the journal to the readers earlier than before. This would also increase the visibility of the articles. We hope the readers will interact more with the authors through the letter to the editor column.

